# Supplementary material for: DNA Vaccine Administered by Cationic Lipoplexes or by In Vivo Electroporation Induces Comparable Antibody Responses against SARS-CoV-2 in Mice
Source: Vaccines (Basel). 2021 Aug 6;9(8):874. doi: 10.3390/vaccines9080874 (PMC8402479; doi:10.3390/vaccines9080874)
Supplement: Supplementary file 1 [file vaccines-09-00874-s001.zip › vaccines-1305818-supplementary.pdf]

Supplementary Figure S1:

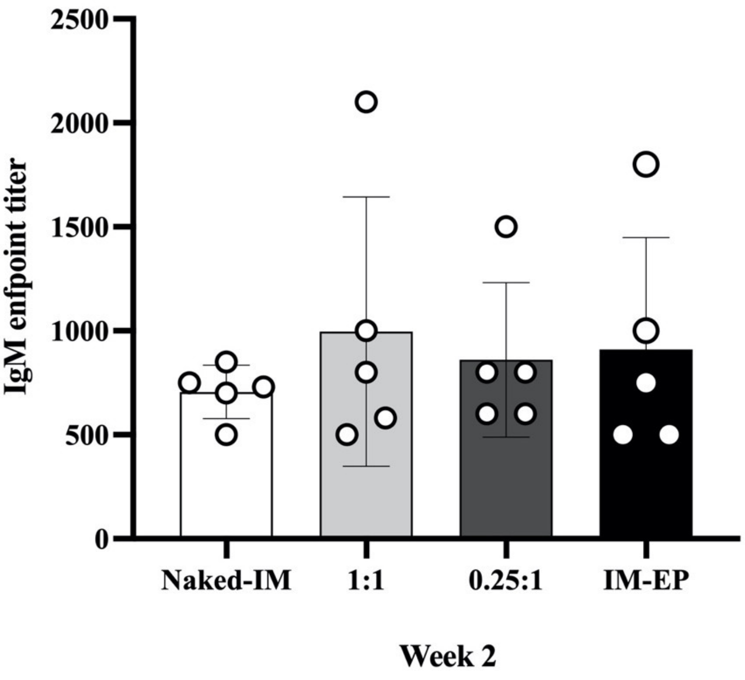

Figure S1: Endpoint titers of SARS-CoV-2 spike-specific IgM analyzed at week 2 in mice immunized intramuscularly with naked pCMVkan-S (IM-naked), pCMVkan-S formulated with DOTAP4 at N/P ratios of 1:1 and 0.25:1 and by using electroporation device (IM-EP). Bars present mean ± SD of the endpoint titers in each vaccination group (n = 5).

Supplementary Figure S2:

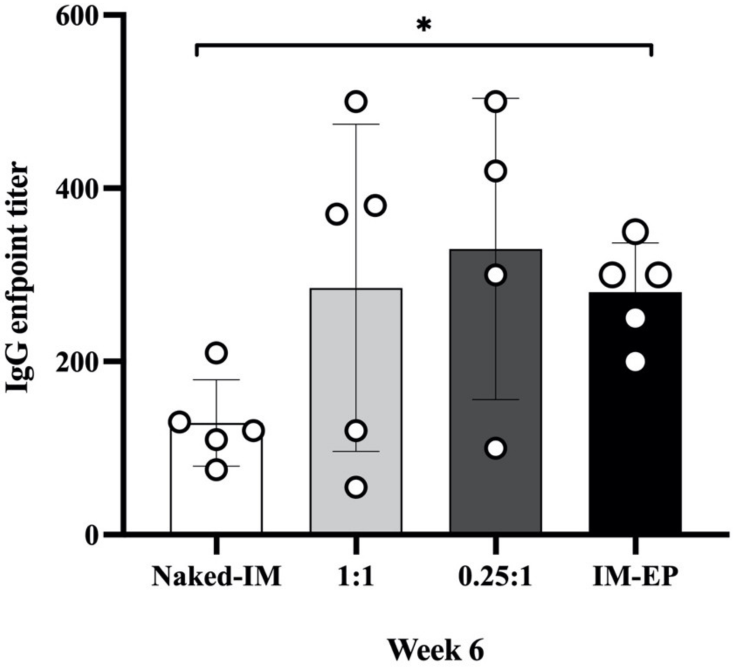

Figure S2: Endpoint titers of SARS-CoV-2 spike-specific IgG analyzed in bronchoalveolar lavage (BAL) at week 6 in mice immunized intramuscularly with naked pCMVkan-S (IM-naked), pCMVkan-S formulated with DOTAP4 at N/P ratios of 1:1 and 0.25:1 and by using electroporation device (IM-EP). Bars present mean ± SD of the endpoint titers in each vaccination group (n = 4-5).

Supplementary Figure S3:

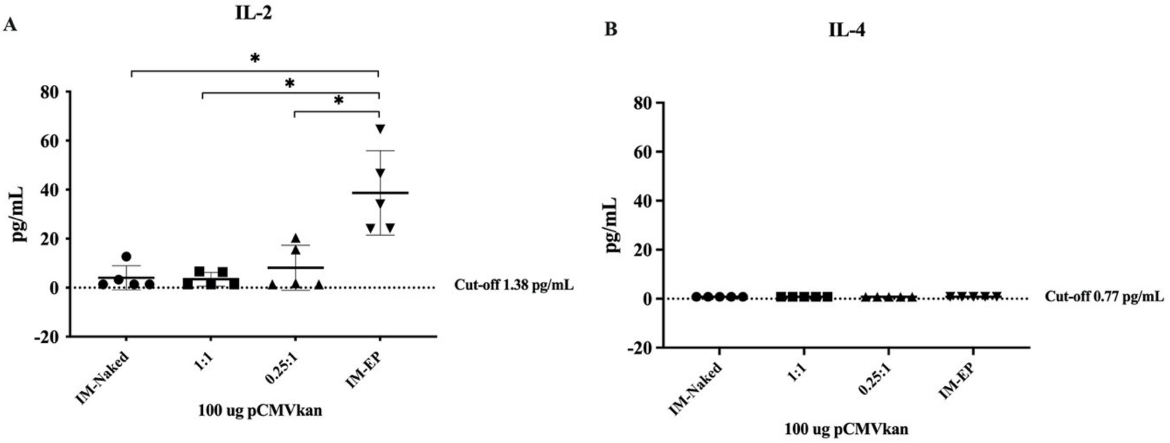

Figure S3: *Ex vivo* IL-2 (A) and IL-4 (B) expression collected from the culture supernatant after splenocytes from S-immunized group were stimulated with spike pooled peptides for 40 h.

Supplementary Figure S4:

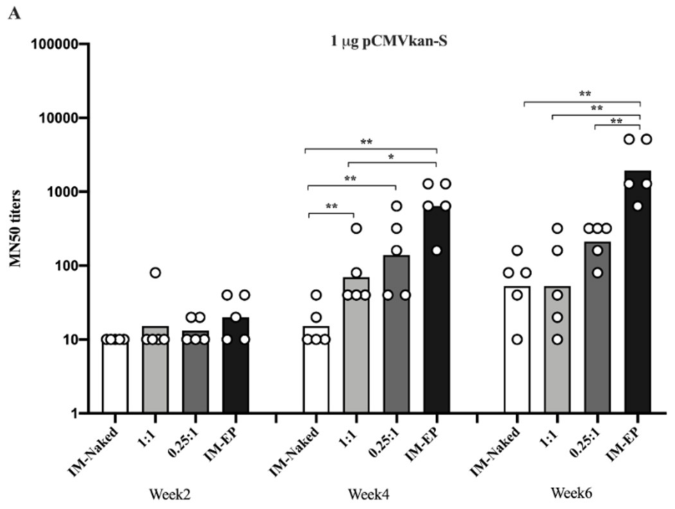

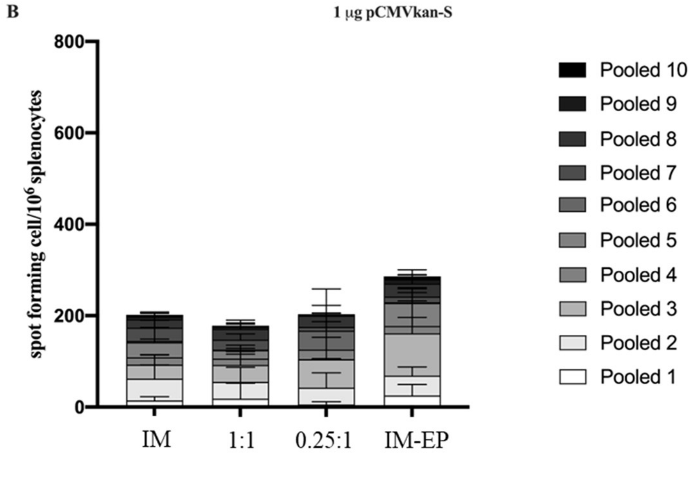

Figure S4: Immune response in mice (N =5) immunized with 1 μg of pCMVkan-S using different immunization strategies including naked pCMVkan-S (IM), pCMVkan-S formulated with DOTAP4 at N/P ratios of 1:1 and 0.25:1 and by using electroporator device (IM-EP). (A) Neutralizing antibody analysis, the titers were measured by microneutralization (MN) assay. Bars represent the geometric mean of the MN50 titer (GMT). (B) SARS-CoV-2 spike-specific T cells responses analyzed by ELISpot. Mice splenocytes were simulated with pooled peptides from S1 (pool 1-5) and S2 (pool 6-10) regions. Each bar represents sum of IFN- γ responses. * and ** indicate $p<0.05$ and $p<0.01$, respectively.

Supplementary Figure S5:

WST-1 assays on RAW 264.7 cells after 24h incubation with lipoplexes

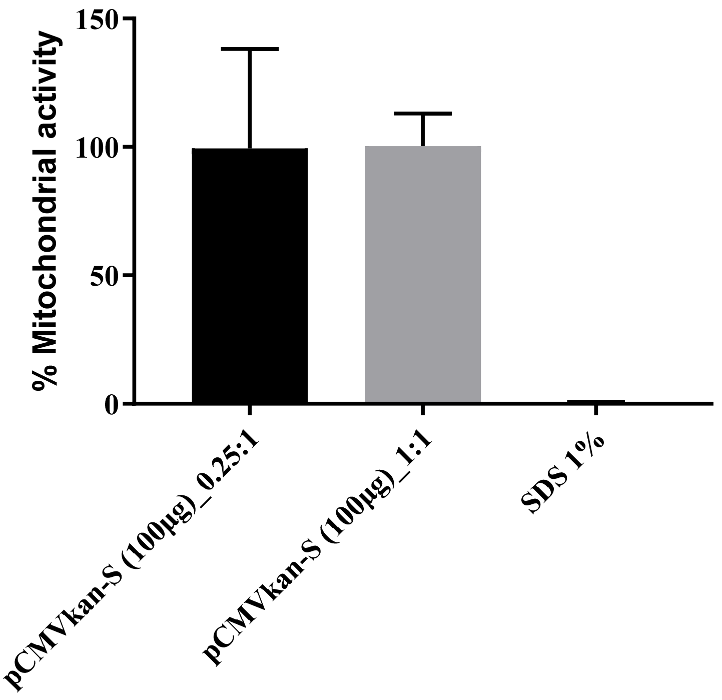

Figure S5: Mitochondrial activity of RAW 264.7 cells after 24 h incubation with lipoplexes at N/P ratios at 0.25:1 and 1:1 for both 100 µg and 1 µg of pCMVkan-S plasmid. Results are normalized to non-treated cells results. SDS 1% was used as a positive control.
